# Supplementary material for: Pyruvate Dehydrogenase Kinase 1 inhibition mediated oxidative phosphorylation enhancement in cartilage promotes osteoarthritis progression
Source: BMC Musculoskelet Disord. 2023 Jul 20;24:597. doi: 10.1186/s12891-023-06585-6 (PMC10357736; doi:10.1186/s12891-023-06585-6)
Supplement: Supplementary file 1 — Additional file 1. [file 12891_2023_6585_MOESM1_ESM.zip › MF.Gsea.1653627316242/GOMF_PHOSPHATIDYLINOSITOL_3_4_BISPHOSPHATE_BINDING.html]

Details for gene set GOMF\_PHOSPHATIDYLINOSITOL\_3\_4\_BISPHOSPHATE\_BINDING[GSEA]

|  || Dataset | 55457\_collapsed\_to\_symbols.cls.cls #OA\_versus\_normal.cls.cls #OA\_versus\_normal\_repos |
| Phenotype | cls.cls#OA\_versus\_normal\_repos |
| Upregulated in class | normal |
| GeneSet | GOMF\_PHOSPHATIDYLINOSITOL\_3\_4\_BISPHOSPHATE\_BINDING |
| Enrichment Score (ES) | -0.49073094 |
| Normalized Enrichment Score (NES) | -1.5662018 |
| Nominal p-value | 0.028571429 |
| FDR q-value | 0.1561847 |
| FWER p-Value | 0.999 |
Table: GSEA Results Summary

  

Fig 1: Enrichment plot: GOMF\_PHOSPHATIDYLINOSITOL\_3\_4\_BISPHOSPHATE\_BINDING      
 Profile of the Running ES Score & Positions of GeneSet Members on the Rank Ordered List

  

| SYMBOL | TITLE | RANK IN GENE LIST | RANK METRIC SCORE | RUNNING ES | CORE ENRICHMENT || 1 | AKT1 | AKT serine/threonine kinase 1 [Source:HGNC Symbol;Acc:HGNC:391] | 1192 | 0.478 | -0.0353 | No |
| 2 | PLEK2 | pleckstrin 2 [Source:HGNC Symbol;Acc:HGNC:19238] | 1525 | 0.425 | -0.0102 | No |
| 3 | RS1 | retinoschisin 1 [Source:HGNC Symbol;Acc:HGNC:10457] | 1682 | 0.401 | 0.0258 | No |
| 4 | NCF1 | neutrophil cytosolic factor 1 [Source:HGNC Symbol;Acc:HGNC:7660] | 2195 | 0.339 | 0.0266 | No |
| 5 | KIF16B | kinesin family member 16B [Source:HGNC Symbol;Acc:HGNC:15869] | 4355 | 0.124 | -0.1262 | No |
| 6 | PLEK | pleckstrin [Source:HGNC Symbol;Acc:HGNC:9070] | 4382 | 0.122 | -0.1136 | No |
| 7 | OBSCN | "obscurin, cytoskeletal calmodulin and titin-interacting RhoGEF [Source:HGNC Symbol;Acc:HGNC:15719]" | 5230 | 0.054 | -0.1729 | No |
| 8 | ADAP2 | ArfGAP with dual PH domains 2 [Source:HGNC Symbol;Acc:HGNC:16487] | 5535 | 0.030 | -0.1929 | No |
| 9 | PHLDA3 | pleckstrin homology like domain family A member 3 [Source:HGNC Symbol;Acc:HGNC:8934] | 6255 | -0.024 | -0.2459 | No |
| 10 | PLEKHA4 | pleckstrin homology domain containing A4 [Source:HGNC Symbol;Acc:HGNC:14339] | 6286 | -0.026 | -0.2450 | No |
| 11 | TTPA | alpha tocopherol transfer protein [Source:HGNC Symbol;Acc:HGNC:12404] | 6796 | -0.063 | -0.2770 | No |
| 12 | RAG2 | recombination activating 2 [Source:HGNC Symbol;Acc:HGNC:9832] | 7462 | -0.115 | -0.3149 | No |
| 13 | WASHC2C | WASH complex subunit 2C [Source:HGNC Symbol;Acc:HGNC:23414] | 7471 | -0.116 | -0.3016 | No |
| 14 | GAB2 | GRB2 associated binding protein 2 [Source:HGNC Symbol;Acc:HGNC:14458] | 7742 | -0.137 | -0.3062 | No |
| 15 | ANXA8 | annexin A8 [Source:HGNC Symbol;Acc:HGNC:546] | 8327 | -0.180 | -0.3300 | No |
| 16 | MAPKAP1 | MAPK associated protein 1 [Source:HGNC Symbol;Acc:HGNC:18752] | 10000 | -0.321 | -0.4214 | No |
| 17 | FCHSD2 | FCH and double SH3 domains 2 [Source:HGNC Symbol;Acc:HGNC:29114] | 10894 | -0.416 | -0.4409 | Yes |
| 18 | PLEKHA1 | pleckstrin homology domain containing A1 [Source:HGNC Symbol;Acc:HGNC:14335] | 10899 | -0.416 | -0.3914 | Yes |
| 19 | PLEKHA2 | pleckstrin homology domain containing A2 [Source:HGNC Symbol;Acc:HGNC:14336] | 10921 | -0.421 | -0.3426 | Yes |
| 20 | HIP1R | huntingtin interacting protein 1 related [Source:HGNC Symbol;Acc:HGNC:18415] | 11717 | -0.535 | -0.3402 | Yes |
| 21 | ARAP3 | "ArfGAP with RhoGAP domain, ankyrin repeat and PH domain 3 [Source:HGNC Symbol;Acc:HGNC:24097]" | 12386 | -0.704 | -0.3078 | Yes |
| 22 | DAPP1 | dual adaptor of phosphotyrosine and 3-phosphoinositides 1 [Source:HGNC Symbol;Acc:HGNC:16500] | 12579 | -0.784 | -0.2288 | Yes |
| 23 | SH3PXD2A | SH3 and PX domains 2A [Source:HGNC Symbol;Acc:HGNC:23664] | 12832 | -1.036 | -0.1243 | Yes |
| 24 | HIP1 | huntingtin interacting protein 1 [Source:HGNC Symbol;Acc:HGNC:4913] | 12858 | -1.081 | 0.0033 | Yes |
Table: GSEA details [plain text format]

  

Fig 2: GOMF\_PHOSPHATIDYLINOSITOL\_3\_4\_BISPHOSPHATE\_BINDING      
 Blue-Pink O' Gram in the Space of the Analyzed GeneSet

  

Fig 3: GOMF\_PHOSPHATIDYLINOSITOL\_3\_4\_BISPHOSPHATE\_BINDING: Random ES distribution      
 Gene set null distribution of ES for **GOMF\_PHOSPHATIDYLINOSITOL\_3\_4\_BISPHOSPHATE\_BINDING**
